# Comparative Assessment of Endoscopic Ultrasound-Guided Biopsies vs. Percutaneous Biopsies of Pancreatic Lesions: A Systematic Review and Meta-Analysis of Diagnostic Performance

**DOI:** 10.3390/jcm13113108

**Published:** 2024-05-25

**Authors:** Daniel Paramythiotis, Eleni Karlafti, Dimitrios Tsavdaris, Konstantinos Arvanitakis, Adonis A. Protopapas, Georgios Germanidis, Leonidas Kougias, Adam Hatzidakis, Christos Savopoulos, Antonios Michalopoulos

**Affiliations:** 1 First Propaedeutic Surgery Department, University General Hospital of Thessaloniki AHEPA, Aristotle University of Thessaloniki, 54636 Thessaloniki, Greece; danosprx@auth.gr (D.P.); tsavdaris@auth.gr (D.T.); amichal@auth.gr (A.M.); 2 Emergency Department, University General Hospital of Thessaloniki AHEPA, Aristotle University of Thessaloniki, 54636 Thessaloniki, Greece; 3 First Propaedeutic Department of Internal Medicine, University General Hospital of Thessaloniki AHEPA, Aristotle University of Thessaloniki, 54636 Thessaloniki, Greece; adoprot@hotmail.com (A.A.P.); chrisavopoulos@gmail.com (C.S.); 4 First Department of Internal Medicine, University General Hospital of Thessaloniki AHEPA, Aristotle University of Thessaloniki, 54636 Thessaloniki, Greece; kostarvanit@gmail.com (K.A.); geogerm@auth.gr (G.G.); 5 Department of Radiology, University General Hospital of Thessaloniki AHEPA, Aristotle University of Thessaloniki, 54636 Thessaloniki, Greece; leonidaskougias@gmail.com (L.K.); adamhatz@auth.gr (A.H.)

**Keywords:** endoscopic ultrasound, percutaneous, biopsy, pancreatic lesions, diagnostic accuracy

## Abstract

**Introduction:** Pancreatic cancer ranks as the fourth deadliest form of cancer. However, it is essential to note that not all pancreatic masses signal primary malignancy. Therefore, it is imperative to establish the correct differential diagnosis, a process further supported by pre-operative biopsy procedures. This meta-analysis aims to compare the diagnostic performance of two minimally invasive biopsy approaches for pancreatic tissue sampling: percutaneous biopsies guided by computed tomography or ultrasound, and transduodenal biopsies guided by endoscopic ultrasound (EUS). **Methods:** A systematic literature search was conducted in the MEDLINE and Scopus databases. The included studies analyzed the diagnostic performance of the two biopsy methods, and they were assessed for risk of bias using the Quality Assessment of Diagnostic Accuracy Studies–2 tool. Statistical analysis was carried out using the RevMan and MetaDisc software packages. **Results:** The statistical analysis of the results demonstrated the superiority of the percutaneous approach. Specifically, the pooled sensitivity, specificity, LR+, LR−and DOR for the percutaneous approach were 0.896 [95% CI: 0.878–0.913], 0.949 [95% CI: 0.892–0.981], 9.70 [95% CI: 5.20–18.09], 0.20 [95% CI: 0.12–0.32] and 68.55 [95% CI: 32.63–143.98], respectively. The corresponding values for EUS-guided biopsies were 0.806 [95% CI: 0.775–0.834], 0.955 [95% CI: 0.926–0.974], 12.04 [95% CI: 2.67–54.17], 0.24 [95% CI: 0.15–0.39] and 52.56 [95% CI: 13.81–200.09], respectively. Nevertheless, it appears that this statistical superiority is also linked to the selection bias favoring larger and hence more readily accessible tumors during percutaneous biopsy procedures. **Conclusions:** Concisely, our meta-analysis indicates the statistical superiority of the percutaneous approach. However, selecting the optimal biopsy method is complex, influenced by factors like patient and tumor characteristics, clinical resources, and other relevant considerations.

## 1. Introduction

Pancreatic cancer ranks as the fourth most fatal cancer in both men and women [1]. Symptoms typically emerge during advanced stages, complicating early detection efforts [2,3]. Additionally, it is crucial to note that not all pancreatic masses indicate primary cancer; they could also stem from conditions like chronic pancreatitis or metastatic neoplasms [4,5]. Hence, it is deemed essential to distinguish between these conditions, including autoimmune pancreatitis, which can be accomplished non-invasively through the detection of serological markers [6,7]. Pancreatic neoplasms encompass a diverse range, including cystic tumors of varying histological origins, behavior, and malignant potential, as well as neoplasms originating from the exocrine portion of the pancreas [8,9].

Concerning cystic pancreatic lesions, they predominantly involve pseudocysts, which are non-neoplastic complications of pancreatitis [10,11,12]. However, it is important to note that cystic pancreatic lesions may also indicate various neoplasms. Specifically, cysts lined with cuboidal epithelial cells can be differentiated and are referred to as serous cystadenoma. These are benign tumors [13]. Additionally, mucinous cystic neoplasms, which primarily arise in the body and tail of the pancreas, exhibit malignant potential [14,15]. Moreover, intraductal papillary mucinous neoplasms are recognized for causing cystic expansion of pancreatic ducts, while this can also indicate solid pseudopapillary neoplasms [16,17,18]. As for exocrine pancreatic cancers, they are either cystic or solid epithelial tumors, with pancreatic ductal adenocarcinomas (PDACs) being the most common among them [19].

In the context of diagnosing pancreatic cancer, there is a wide array of techniques and methodologies that can be employed to identify the disease. While biopsies are not required for patients with characteristic imaging findings and resectable tumors, most patients will require histological confirmation due to having a disease that is unresectable or having an inconclusive diagnosis. Minimally invasive biopsy procedures can be conducted with the guidance of computed tomography (CT) or ultrasound (US) imaging. These two techniques utilize the percutaneous route to approach the pancreas. As for CT guidance, it delineates optimal visualization of the anatomical region, allowing visualization of the surrounding vascular and other sensible structures, is not affected by the presence of intestine air and is particularly suitable for elderly patients with lower capability to cooperate [20,21] However, the use of CT guidance is characterized by radiation exposure, although usually not of a high level. To counter this, it has been demonstrated that the use of contrast agents reduces the duration of the procedure and therefore the duration of radiation exposure. Furthermore, contrast agent administration aids in distinguishing necrotic areas from viable areas, contributing to correct biopsy sampling [20,21].

As for US-guided biopsies, they can be performed with higher variability of access routes due to the variety of projections which can be chosen. To ensure greater safety and avoid injury to non-target structures, the upper left quadrant is preferred. Also, to avoid major blood vessels, color dopplers are valuable tools. Moreover, two types of probes can be used for performing biopsies: those equipped with lateral support and those featuring discontinuous crystals and central support. The advantages over CT-guided biopsies primarily revolve around greater accessibility, shorter intervention time, absence of radiation exposure, and lower cost [22,23,24].

Regrettably, when samples are procured under CT or US guidance, the needle frequently needs to traverse intervening structures within the peritoneal cavity. This results in a high rate of false negative diagnoses, diminishing the effectiveness of the CT- or US-guided approach in the preoperative evaluation of patients. The so-called “co-axial” technique uses a larger guiding needle through which the cutting needle can be repeatedly used without the risk of tumor seeding. Such safe access can be found even through the left liver lobe, with the extra possibility of embolizing the transhepatic route by inserting short pieces of Gelfoam through the co-axial needle while retrieving it. Another technique to access deep pancreatic biopsy targets is to go through a posterior paravertebral route, with the patient in the prone position. Sometimes, if no free route is found, the creation of a safe pathway through other anatomic structures can be achieved with the so-called “artificial widening technique” [25]. With this technique, it is possible to push away vital structures by injecting saline through a fine 20–21-gauge needle, and so create a free straight way towards the region of interest.

The risk of transgressing non-target structures can be also eliminated by taking a biopsy under endoscopic ultrasound (EUS) guidance. This can be classified into two categories, radial and linear, with the first one providing views similar to CT guidance and the second one similar to US guidance. However, radial EUS is not a suitable technique for performing pancreatic biopsies. The intervention is performed under deep sedation. Afterward, the tissue is taken, which can be conducted through fine needle aspiration (FNA), usually using 19–25-gauge caliber needles [26,27,28]. These techniques allow for better visualization and approximation of the tissue under examination, as compared with CT- or US-guided biopsies. Based on these considerations, guidelines advocate for the utilization of EUS-directed biopsies for patients requiring a histopathological diagnosis [29,30].

It is therefore evident that numerous methods exist for obtaining histological preparations, as well as for diagnosing pancreatic cancer. The methods have varying performance levels and exhibit specific advantages and disadvantages. Hence, in this systematic review and meta-analysis, our objective is to analyze and compare the diagnostic efficacy between the percutaneous approaches and EUS in diagnosing pancreatic cancer. This comparison enhances clinical decision-making by identifying the biopsy method with superior diagnostic accuracy, potentially reducing unnecessary procedures and expediting appropriate treatment initiation for patients with pancreatic lesions.

## 2. Materials and Methods

### 2.1. Study Protocol and Guidelines

This systematic review and meta-analysis was conducted in accordance with the Preferred Reporting Items for Systematic Reviews and Meta-Analyses (PRISMA) guidelines [31]. This systematic review and meta-analysis is registered in the Open Science Framework (OSF), with the registration number DOI 10.17605/OSF.IO/9W4XJ.

### 2.2. Eligibility Criteria

The eligibility criteria for this systematic review and meta-analysis are outlined and were established using the PICO (population, intervention, comparison, and outcomes) framework. Studies were considered for inclusion if they met the following criteria:

- Adult patients who have undergone a pancreatic biopsy, whether for suspicion of pancreatic cancer, evaluation of pancreatic lesions, or any other reason.
- Biopsy methods encompassing EUS-guided biopsies and percutaneous biopsies.
- Comparison between the two methods.
- Diagnostic accuracy of each method.

Reviews, letters, comments, surveys, meta-analyses, pilot studies, conference abstracts and case reports were excluded. Articles not written in the English language were also excluded from this systematic review and meta-analysis. Hence, this systematic review and meta-analysis exclusively considered original articles in which a direct comparison of the diagnostic accuracy and performance of both EUS and percutaneous guided biopsies was performed.

### 2.3. Information Sources, Search Strategy and Selection Process

The search was conducted in the online databases PubMed and Scopus using the search terms (“pancreatic biopsy” OR “pancreatic lesion biopsy”) AND (“pancreatic lesions” OR “pancreatic neoplasms” OR “pancreatic cancer») AND (“diagnostic accuracy” OR “diagnostic yield” OR “sensitivity” OR “specificity”) AND (“biopsy techniques” OR “fine-needle aspiration” OR “endoscopic ultrasound-guided biopsy”). No search filters or time limits were applied. The selection process involved two independent reviewers (D.T. and E.K.) who assessed the eligibility of studies for inclusion in this systematic review and meta-analysis based on title, abstract and full-text evaluation. Any disagreements were resolved by a third reviewer (Κ.A.).

### 2.4. Data Collection Process and Data Items

Data extraction was conducted by one reviewer (D.T.), with another reviewer (E.K.) independently verifying the results. The data were extracted into a standardized Excel form, without the assistance of any automation tools. Key information extracted from the reviewed studies included the main characteristics of each study, such as author and year of publication, location, total number of tissue samples, study type, and diagnostic parameters like sensitivity, specificity, negative predictive value (NPV), positive predictive value (PPV) and accuracy for each biopsy technique. Any diagnostic parameter not initially provided was computed using true positive (TP), true negative (TN), false positive (FP) and false negative (FN) values, using RevMan 5.4 software whenever feasible. In this context, TP signifies cases where the test accurately identifies the presence of disease, while TN denotes cases where the test accurately identifies the absence of disease. Conversely, FP indicates cases where the test inaccurately suggests the presence of disease, and FN indicates cases where the test inaccurately suggests the absence of disease.

### 2.5. Study Risk of Bias Assessment

The risk of bias in the included studies was assessed using the Quality Assessment of Diagnostic Accuracy Studies–2 (QUADAS-2) tool, which comprises four main domains: patient selection, index test, reference standard, flow and timing [32,33], and its extension for comparative reviews (QUADAS-C) [34].

### 2.6. Statistical Analysis

The statistical analysis was conducted using RevMan Software by Cochrane (v.5.3) and Meta-Disc [35]. Meta-analysis of the sensitivity and specificity was carried out utilizing a Der Simonian and Laird random-effects model, as it suited our analysis given the heterogeneity observed. Heterogeneity among studies was assessed using the I^2^ and the Cochran Q statistic. One reviewer (D.T.) performed the statistical analysis, and another reviewer (K.A.) independently verified the results. A probability level of 0.05 was considered statistically significant for all tests (excluding heterogeneity). In the studies included in this systematic review and meta-analysis, further subgrouping was not conducted based on uniform characteristics; for example, based on demographic characteristics or tumor characteristics. Therefore, statistical analysis based on subgroups was not feasible.

## 3. Results

### 3.1. Study Selection

**Figure 1:**
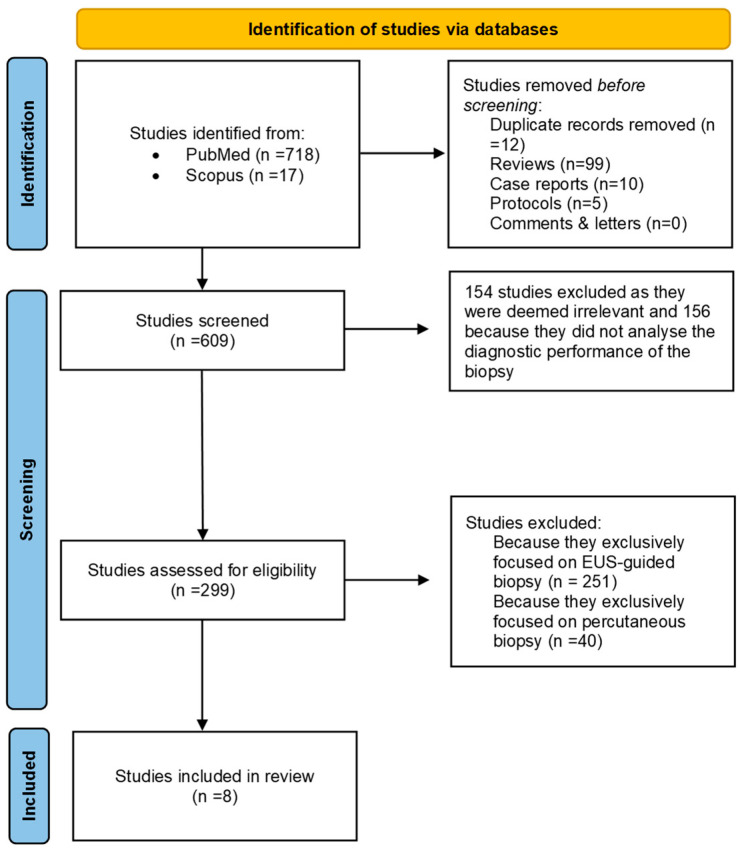
PRISMA 2020 flow chart. This diagram illustrates the process followed for the collection and selection of studies used in our systematic review and meta-analysis.

A total of 735 studies were identified using the specified keywords [(“pancreatic biopsy” OR “pancreatic lesion biopsy”) AND (“pancreatic lesions” OR “pancreatic neoplasms” OR “pancreatic cancer”) AND (“diagnostic accuracy” OR “diagnostic yield” OR “sensitivity” OR “specificity”) AND (“biopsy techniques” OR “fine-needle aspiration” OR “endoscopic ultrasound-guided biopsy”)], with 718 retrieved from MEDLINE and 17 from Scopus. Among these, 126 were excluded due to duplication or being letters, case reports, reviews or comments. Out of the 609 studies initially identified, 154 were deemed irrelevant and excluded from further consideration. Additionally, 156 studies, while mentioning biopsy for pancreatic cancer, did not analyze the diagnostic performance of biopsy methods and were consequently excluded from the review. Subsequently, 299 studies were screened for eligibility, out of which eight met the inclusion criteria. These selected studies analyzed the diagnostic accuracy, sensitivity, specificity, PPV and NPV of percutaneous and EUS-guided biopsy techniques. The selection process for the studies included in this systematic review and meta-analysis is depicted in a PRISMA 2020 flow chart (Figure 1), illustrating all stages of selection from initial screening to final inclusion.

### 3.2. Study Characteristics

In the eight studies included in this systematic review and meta-analysis [36,37,38,39,40,41,42,43], a total of 2711 tissue samples were obtained for biopsy for the diagnosis of pancreatic cancer. The biopsies were obtained by EUS-FNA and by CT- or US-guided FNA or by US-guided core needle biopsy (CNB). Of these, 1415 were obtained using EUS, while the remaining 1323 were obtained using the percutaneous route. The majority of samples were obtained in two of these eight studies: Volmar et al., 2005 (1050) and Chai et al., 2023 (1074). It is crucial to note that the overarching findings primarily stem from these two substantial retrospective studies. These studies portray a heterogeneous patient distribution, with Volmar et al. predominantly employing EUS and reporting superior outcomes, whereas the Chai et al. study predominantly utilized percutaneous methods and observed comparable excellence. Such disparity potentially underscores the specialized focus of each respective center. Six of these eight studies were observational retrospective cohort (ORCS) studies, while the remaining two were clinical studies, one of which was a randomized controlled trial (RCT), with 41 samples taken via EUS and 43 via percutaneous biopsy. Diagnostic accuracy varies between studies and between techniques. The presence of both randomized controlled trials and ORCS raises the risk of bias. Regarding the use of EUS, the diagnostic accuracy varies from 73% to 89.8%. While regarding the biopsy through the percutaneous route, the diagnostic accuracy varies from 75.6% to 95.2%. The main characteristics of the studies are detailed in Table 1.

### 3.3. Findings of the Studies

#### 3.3.1. Demographic Characteristics and Tumor Localization in Pancreatic Cancer Patients

In the studies reporting the demographic characteristics of patients with pancreatic cancer, a greater frequency was observed in the male gender, as in all studies they constitute the majority of patients, as well as greater frequency of ages over 60 years. It also becomes clear that there is a greater preponderance of cancer occurrences in the head-uncinate process, then the neck and body and finally in the tail of the pancreas. These data are observed both in patients who underwent EUS and in patients who underwent percutaneous biopsy. Finally, a large variation is also observed in the lesion diameters, which vary from 2.6 ± 0.1 to 3.66 + 1.31 centimeters, with a slightly larger diameter in patients who underwent percutaneous interventions. Demographic characteristics and tumor localization in pancreatic cancer patients are listed in Table 2.

#### 3.3.2. Biopsy Pathology Analysis in Pancreatic Lesions

**Figure 2:**
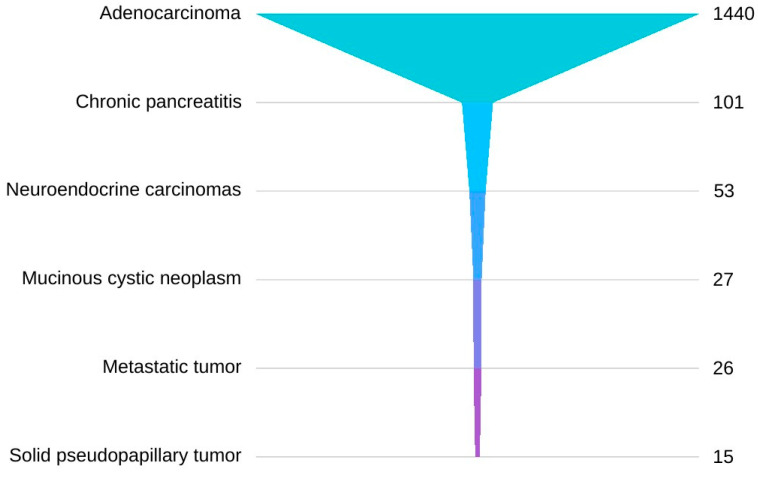
Distribution of pathological types of pancreatic cancer.

The findings derived from these systematic review and meta-analysis align with the epidemiological data, especially in relation to the pathological subtype of pancreatic cancer. It has been consistently observed that adenocarcinomas account for approximately 90% of pancreatic cancer cases [44]. This assertion finds further reinforcement in the studies encompassed within this systematic review and meta-analysis, wherein a total of 1440 cases of adenocarcinoma were documented, constituting 87.32% of the total of 1649 reported pathological diagnoses. Of the remaining 224 tissue samples, 101 confirmed the existence of chronic pancreatitis as the cause, while 53 confirmed neuroendocrine carcinomas. Mucinous cystic neoplasms (n = 27), metastatic tumors (n = 26) and solid pseudopapillary tumors (n = 15) appeared less frequently. However, it is worth noting that other diagnoses, such as adenosquamous carcinomas, gastrointestinal stromal tumors (GIST) in the duodenum, lymphomas and others, were also reported with lower frequency. Pancreatic biopsy findings regarding pathological types of pancreatic cancer are depicted in Figure 2.

#### 3.3.3. Diagnostic Performance of Percutaneous and Endoscopic Ultrasound-Guided Biopsies in Pancreatic Lesions

Regarding the diagnostic performance of the two methods, this can be expressed by using parameters such as sensitivity and specificity, as well as NPV and PPV. Both EUS and CT/US showed high rates of sensitivity and specificity as well as NPV and PPV. For EUS, the sensitivity ranged from 42% to 89.7% and the specificity from 44.4% to 100%, with four studies stating 100% [36,38,39,42]. Conversely, for CT/US sensitivity ranged from 62% to 95.3%, and specificity ranged from 85.7% to 100% across seven studies, with six studies stating 100% [36,37,38,39,40,42]. These percentages indicate that both biopsy techniques have the ability to accurately diagnose both TP and TN cases. In addition, the two techniques can indicate a higher probability of a positive test result being true with significant success, as they note high PPV rates, however, they seem to lag behind in NPV, resulting in a higher probability of a negative test result being false. The two largest studies yielded contradictory results, with one indicating a higher diagnostic accuracy for EUS [37] and the other for CT/US [41]. Nonetheless, the precision of the results was very similar between the two techniques.

The selection of biopsy needle size is also a critical factor influencing both diagnostic accuracy and the incidence of intraoperative biopsy complications. It appears that employing a larger needle size is associated with statistically higher diagnostic accuracy, albeit with an increased risk of bleeding complications and procedural discomfort. Among the eight studies included in this systematic review and meta-analysis, six [36,38,39,41,42,43] utilized a 22-gauge needle for EUS-guided biopsies, with Sur et al. being the sole study employing a 25-gauge needle. In the case of percutaneous biopsies, 20- and 22-gauge needles were utilized in all studies except for Sur et al. and Chai et al. [40,41], who employed 18-gauge needles. Notably, Volmar et al. [37] did not specify the needle size utilized for biopsy acquisition. Consequently, a notable discrepancy in needle diameter is evident, primarily in the study by Sur et al., highlighting the need for enhanced transdermal diagnostic accuracy [40]. The percentages of sensitivity, specificity, NPV and PPV are listed in Table 3.

### 3.4. Quality Assessment

QUADAS-2 and its extension for comparative reviews (QUADAS-C) was employed to evaluate the quality of the studies, assessing each domain for risk of bias, with the first three domains also scrutinized for concerns regarding applicability. This tool aids in identifying bias and enhancing the transparency of the systematic review and meta-analysis.

**Figure 3:**
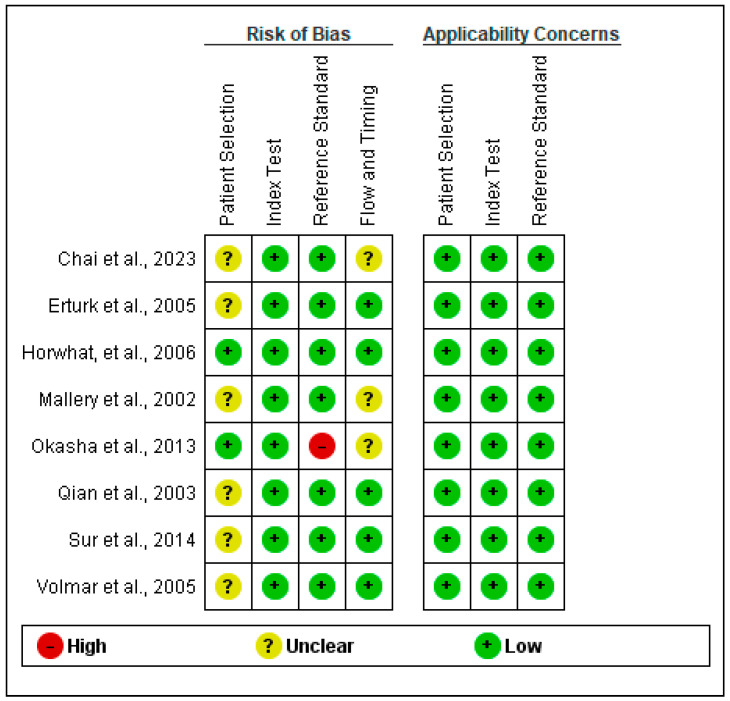
Risk of bias and applicability concerns summary [36,37,38,39,40,41,42,43].

**Figure 4:**
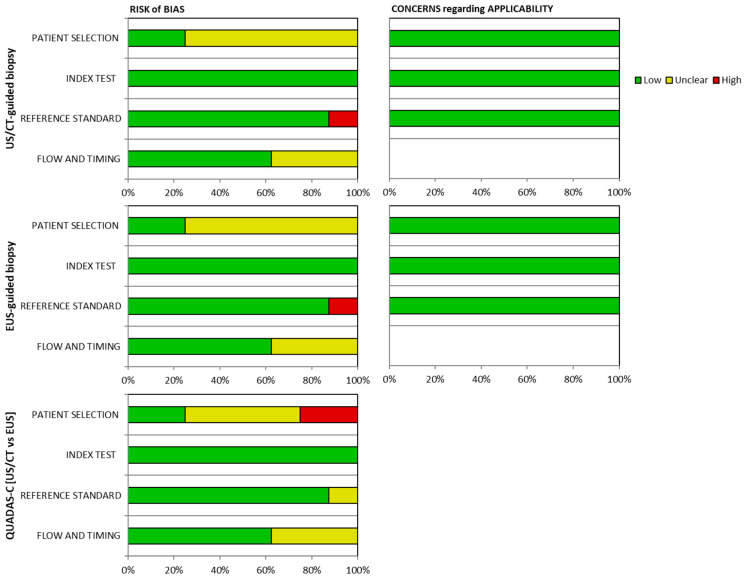
Risk of bias and applicability concerns graph.

Specifically, only two studies were found to have no risk of bias regarding patient selection. In the remaining studies either a consecutive or random allocation of patients enrolled was not achieved, or a case-control design was not avoided, or these characteristics were not described in the methodology. Okasha et al. [43] also rated as high risk in terms of the reference standard, as it is not described in the methodology if the reference standard results were interpreted without knowledge of the results of the index tests. Finally, three studies have unclear bias in the flow and timing section as they did not state if there was an appropriate interval between their index test and reference standard and if all patients received the same reference standard. The outcomes of applying this tool to the analyzed studies are presented in Figure 3 and Figure 4 below.

### 3.5. Meta-Analysis Results

**Figure 5:**
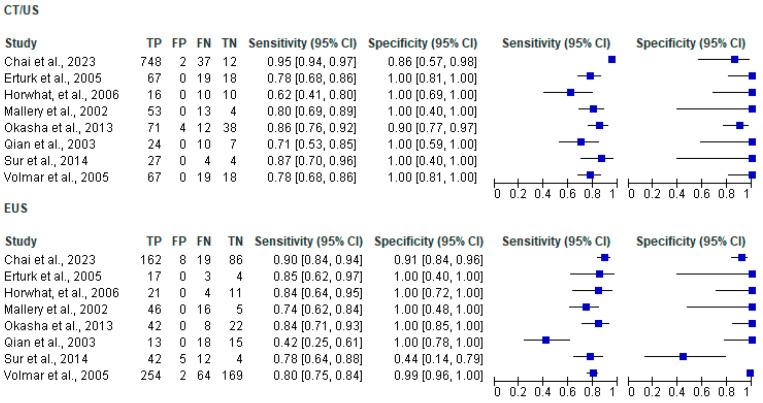
Forest plots displaying pooled sensitivity and specificity are depicted, with true positive (TP), false positive (FP), false negative (FN) and true negative (TN) values for each study, along with the calculated sensitivities and specificities. The pooled sensitivity for CT/US was found to be 0.896 [95% CI: 0.878–0.913] (heterogeneity chi-squared = 83.27, *p* = 0.000, Ι^2^ = 91.6%), while for EUS it was 0.806 [95% CI: 0.775–0.834] (heterogeneity chi-squared = 35.82, *p* = 0.000, Ι^2^ = 80.5%). Regarding specificity, the pooled value for CT/US was 0.949 [95% CI: 0.892–0.981], (heterogeneity chi-squared = 9.28, *p* = 0.233, Ι^2^ = 24.6%), whereas for EUS it was 0.955 [95% CI: 0.926–0.974] (heterogeneity chi-squared = 33.28, *p* = 0.000, Ι^2^ = 79%) [36,37,38,39,40,41,42,43].

**Figure 6:**
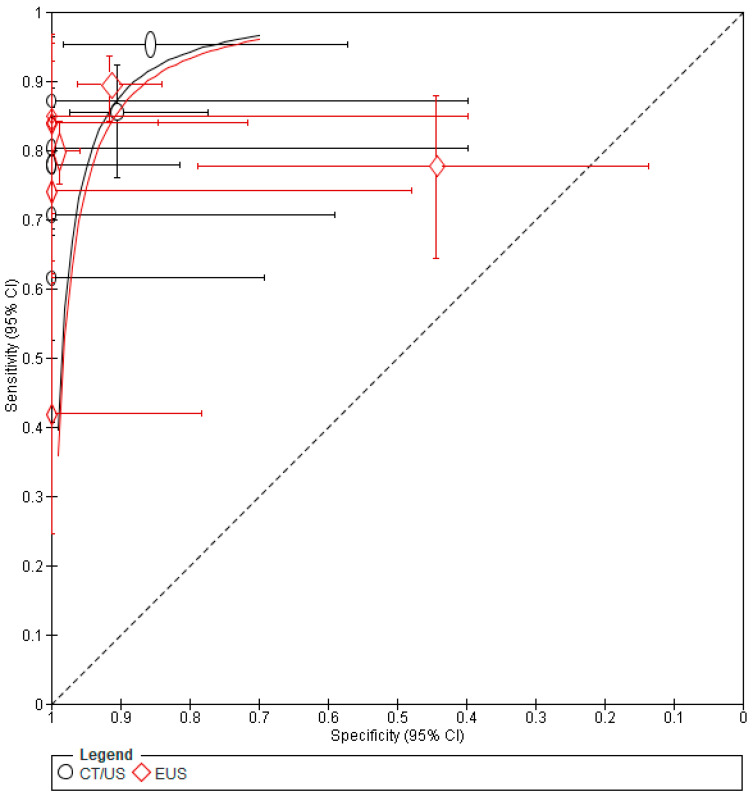
SROC plot. Regarding the area under the curve (AUC), the percutaneous approach yielded a value of 0.9543 (standard error = 0.0139), while the EUS-guided biopsy approach yielded a value of 0.9019 (standard error = 0.0388). These findings suggest the superior performance of the percutaneous approach.

The results of the meta-analysis are shown in the Figure 5 below. The pooled sensitivity for CT/US was found to be 0.896 [95% CI: 0.878–0.913], while for EUS it was 0.806 [95% CI: 0.775–0.834]. These results indicate the superiority of CT/US percutaneous biopsies over EUS. Therefore, percutaneous biopsy is considered more capable of correctly identifying individuals with pancreatic cancer. Conversely, regarding the specificity of biopsy techniques, the EUS approach shows its superiority. Specifically, the pooled value for CT/US was 0.949 [95% CI: 0.892–0.981], whereas for EUS it was 0.955 [95% CI: 0.926–0.974]. Nevertheless, the remarkable sensitivity and specificity exhibited by both techniques underscore their comparability and efficacy in diagnosing pancreatic lesions, reaffirming their utility as valuable diagnostic modalities. The SROC plot is shown in Figure 6.

**Figure 7:**
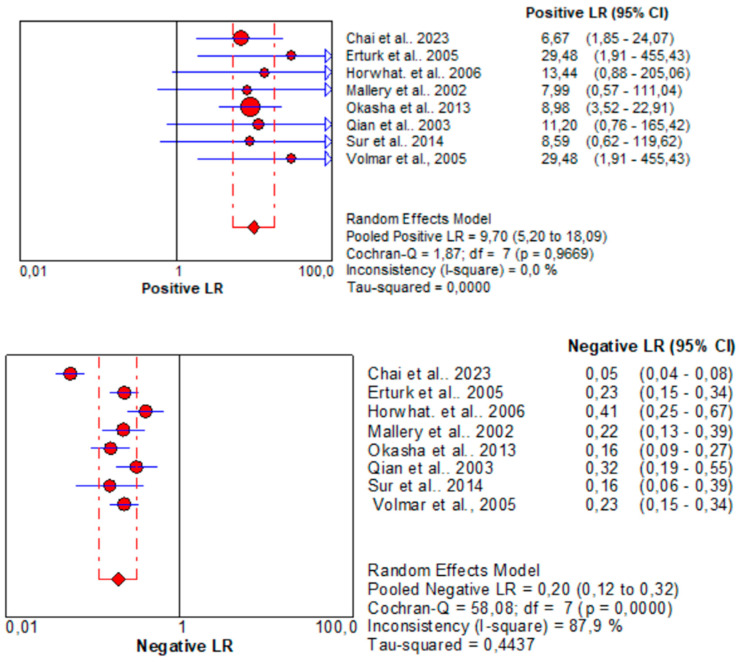
Likelihood ratios for CT/US-Guided biopsy in pancreatic cancer diagnosis. The red circles denote the outcomes of each individual study, while the rhombus serves as the pooling symbol [36,37,38,39,40,41,42,43].

**Figure 8:**
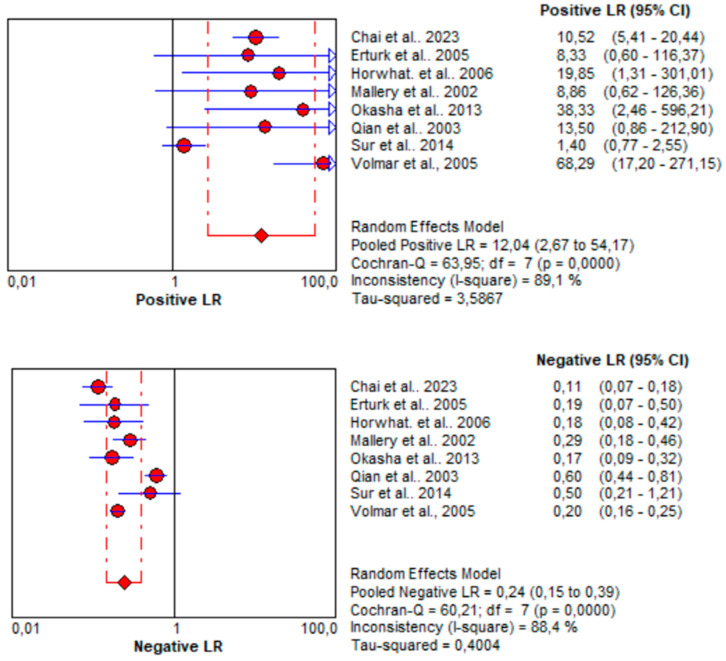
Likelihood ratios for EUS-guided biopsy in pancreatic cancer diagnosis. The red circles denote the outcomes of each individual study, while the rhombus serves as the pooling symbol [36,37,38,39,40,41,42,43].

Subsequently, the parameters associated with the positive (LR+) and negative (LR−) likelihood ratios were analyzed. Regarding EUS, LR+ and LR−, 12.04 [95% CI: 2.67–54.17] and 0.24 [95% CI: 0.15–0.39] were calculated, respectively. Those values for CT/US-guided biopsy are 9.70 [95% CI: 5.20–18.09] and 0.20 [95% CI: 0.12–0.32], respectively. The results of the statistical analysis reveal that CT/US-guided biopsy shows LR+ values within the 5–10 range, indicating moderate evidence in favor of the condition when the test result is positive, while EUS-guided biopsy surpasses this range, suggesting stronger evidence in favor of the condition when the test result is positive. Furthermore, the value of LR− for CT/US-guided biopsy falls within the range of 0.1 to 0.2, indicating moderate evidence against the condition when the test result is negative. Conversely, EUS-guided biopsy exceeds this range slightly, highlighting the superiority of CT/US in this aspect. LRs for CT/US-guided biopsy are depicted in Figure 7, while for EUS-guided biopsy in Figure 8.

**Figure 9:**
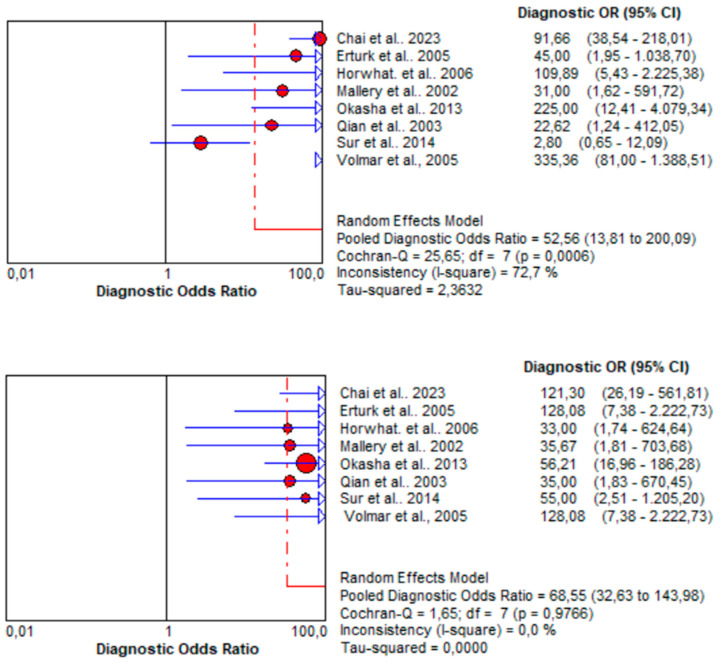
Comparison of pooled diagnostic odds ratios (random effects models) for endoscopic ultrasound (EUS) (top image) and computed tomography/ultrasound (CT/US) (bottom image) biopsy techniques in pancreatic cancer diagnosis. The red circles denote the outcomes of each individual study [36,37,38,39,40,41,42,43].

Finally, a statistical analysis was conducted to compare the summary diagnostic odds ratios (DORs) for EUS and CT/US biopsy techniques. Higher DOR values indicate better discriminatory capabilities for the test, with values greater than one indicating a positive association between the test result and the presence of the condition. In this analysis, a clear superiority of the DORs for CT/US biopsy techniques was observed, as they yielded a pooled DOR of 68.55 [95% CI: 32.63–143.98], contrasting with the pooled DOR of EUS, which scored 52.56 [95% CI: 13.81–200.09]. The comparison of pooled diagnostic odds ratios for EUS and CT/US biopsy techniques in pancreatic cancer diagnosis is illustrated in Figure 9.

## 4. Discussion

In this systematic review and meta-analysis, eight studies [36,37,38,39,40,41,42,43] were included, analyzing and comparing diagnostic accuracy between CT or US percutaneous biopsy of pancreatic cancer and EUS-guided biopsy. The majority of them were ORCS; however, two out of the eight were clinical studies. These studies show high heterogeneity, but they all reach the same conclusion; that both percutaneous biopsy and EUS-guided biopsy are effective in diagnosing pancreatic cancer; however, not with the same success. In two of the seven studies reporting diagnostic accuracy, EUS-guided biopsy was found to be more effective, while in the remaining five, percutaneous biopsy showed superiority. There was a large variation in the diagnostic accuracy of 60.9–89.8% using EUS, in comparison to 75.6–95.2% using percutaneous biopsy.

Using the parameters of TP, TN, FP and FN, a statistical analysis was conducted to determine the pooled sensitivity, specificity and positive and negative LR, as well as DOR. For EUS-guided biopsy the pooled sensitivity, specificity, LR+, LR− and DOR were calculated at 0.806 [95% CI: 0.775–0.834], 0.955 [95% CI: 0.926–0.974], 12.04 [95% CI: 2.67–54.17], 0.24 [95% CI: 0.15, 0.39] and 52.56 [95% CI: 13.81–200.09], respectively. The corresponding results for percutaneous biopsy were 0.896 [95% CI: 0.878–0.913], 0.949 [95% CI: 0.892–0.981], 9.70 [95% CI: 5.20–18.09], 0.20 [95% CI: 0.12, 0.32] and 68.55 [95% CI: 32.63–143.98], respectively. Therefore, in terms of sensitivity, the percutaneous approach emerges as being more capable of correctly identifying individuals with pancreatic cancer, but, due to lower pooled specificity, not to correctly identify individuals without the condition. Furthermore, with the utilization of percutaneous biopsy, there is a higher likelihood of stronger evidence against but not in favor of the condition whenever the test result is negative or positive, respectively, as indicated by the pooled negative and positive LRs. Finally, percutaneous biopsy demonstrated better discriminatory ability between the test result and the presence of the condition, as the pooled DOR was higher.

Despite the statistical superiority of the percutaneous approach over EUS suggested by this systematic review and meta-analysis, no definitive conclusion can be drawn due to limitations of the included studies. Specifically, the included studies seem to have a bias towards selecting larger tumors for percutaneous biopsies, making tissue acquisition easier and more affordable, thus tipping the statistical favor in favor of percutaneous approaches. As such, comprehensive interpretation of these findings demands consideration of these factors as well as their implications for clinical practice. In summary, regarding the clinical implications of the meta-analysis, the findings suggest that both percutaneous and endoscopic biopsy approaches can be utilized in large pancreatic masses. However, the endoscopic approach appears to be more suitable for small pancreatic tumors, making it the preferred option in such cases. Hence, guidelines are progressively advocating for the utilization of EUS biopsy due to its capability of detecting smaller tumors with equivalent diagnostic performance, superior visualization of lesions and fewer associated complications. These advantages are reflected in the guidelines, which recommend EUS as the primary diagnostic tool in most scenarios.

The success of each intervention appears to be influenced by a multitude of factors. These factors may include age, sex, size and location of the lesion, involvement of a cytologist during tissue sampling and various other considerations. In particular, Mallery et al. commented on the effect of these factors in terms of the overall diagnostic accuracy of the methods, without distinguishing and comparing between them [39]. The results showed that being aged over 65 and male were associated with better results. In addition, higher diagnostic accuracy is achieved when biopsies are accompanied by the participation of a cytologist during tissue sampling. Regarding the location of the lesion, it appears that diagnostic accuracy is higher for tumors located in the tail or body of the pancreas as compared to those in the head, despite the head being the most common location for pancreatic tumors [45].

The experience of both gastroenterologists and radiologists in performing the biopsy is also another factor affecting diagnostic accuracy. However, in the included studies, those which reported the experience of healthcare professionals [40,41] reported the extensive experience of the personnel in performing the biopsy, significantly limiting its effect on the results of the studies. The remaining studies, however, did not report their experience of the healthcare professionals in performing the biopsy.

The diagnostic accuracy of biopsy methods appears to be significantly impacted by the presence of chronic pancreatitis. Specifically, the sensitivity of the methods has a notable decline, potentially dropping by as much as 20% in patients with chronic pancreatitis. However, it is worth highlighting that the presence of chronic pancreatitis does not seem to have a substantial effect on specificity and PPV [46,47].

Regarding pancreatic lesion size, it seems that it exerts a significant influence on diagnostic accuracy, having an important role in the choice of the appropriate intervention [48]. In general, a size greater than 3 cm seems to increase the diagnostic accuracy of all biopsy methods [39]. Volmar et al. [37] estimated that the sensitivity showed an increase from 80% [95% CI: 44.9–100] to 84.2% [95% CI: 72.6–95.8] in percutaneous biopsy when the size of the lesion increased from less than 2 cm to greater than 3 cm. However, regarding EUS, Volmar et al. [37] observed a reduction from 86.5% [95% CI: 75.5–97.5] to 83.1% [95% CI: 76.6–89.5] in the corresponding size, a reduction that was also confirmed by the data of Erturk et al. [36], who found a reduction from 93.8% [95% CI: 71.7–98.9] to less than 50% [95% CI: 15–85] for lesions greater than 3 cm. However, these results are accompanied by logistic regression. In the majority of studies, EUS was used in slightly smaller lesions than the percutaneous approach.

As for the accuracy of EUS-guided biopsy, it appears to be influenced by a multitude of additional factors. Needle size is one such factor. However, its influence on the ultimate outcome does not appear to hold significant clinical importance. Data indicate that the variance in diagnostic accuracy between needle gauges ranging from 25-gauge to 22 is approximately 10%. However, the impact of these factors may vary depending on the characteristics of the lesion and the expertise of the endoscopist. On the contrary, it seems that the need for a higher number of passes in the use of the 25-gauge is important, as the smaller size of the needle also means collecting a smaller tissue sample [49,50,51,52,53].

The utilization of rapid on-site evaluation (ROSE) in EUS-FNA procedures has been demonstrated to significantly streamline procedural workflow. ROSE involves promptly analyzing tissue samples acquired during the biopsy, typically requiring the presence of a cytotechnologist or pathologist to assess the sample immediately upon receipt. While the data on its effectiveness are somewhat mixed, ROSE offers several benefits and contributes to enhancing the performance of EUS-FNA. It has been associated with a reduced need for repeat procedures and provides real-time guidance during the biopsy process. Furthermore, evidence suggests that ROSE may improve the sensitivity and specificity of the biopsy, resulting in more accurate diagnoses [54,55,56,57,58,59,60]. An alternative approach to ROSE is macroscopic on-site evaluation, which can serve as an indicator of specimen adequacy and potentially enhance the diagnostic yield of EUS-FNA. However, the available evidence on its efficacy is limited [61,62,63,64].

EUS-guided sample collection can be accomplished using two distinct techniques. While the standard approach is EUS-FNA for tissue sampling during EUS procedures, it appears that EUS fine-needle biopsy (EUS-FNB) also offers certain advantages over FNA. EUS-FNB involves obtaining tissue cores and is associated with fewer needle passes, shorter procedure time and excellent histological yield, all at comparable cost [65,66,67,68,69].

Finally, concerning EUS guidance, the technique employed for tissue sample acquisition also significantly impacts diagnostic accuracy. The “fanning” technique is regarded as highly reliable, as it collects tissue for biopsies from various locations. Consequently, it substantially enhances the diagnostic yield to 86%, compared to the 50% yield achieved by the standard technique [70].

The technical success of both methods seems to be very high. Technical success is defined as the attainment of the region of interest and the procurement of a specimen from the pancreatic lesion [71]. In case of technical failure of one technique, it is possible to obtain a biopsy through the other technique. However, in many cases the biopsies showed insufficient sampling. In most of the eight studies included in this systematic review and meta-analysis, the majority of inadequate sampling was detected during EUS-guided biopsies. Chai et al. [41] quantified the factors that influence the success of the intervention or lead to inconclusive pathological biopsies, repeat biopsies and inaccurate diagnoses. Regarding inconclusive pathological biopsy results, several factors appear to be associated. These include the size of the lesion, particularly when it is less than 2 cm, the method of biopsy via EUS, as well as the hypoechoic appearance and non-pancreatic PDAC diagnosis. The latter two factors are also linked to the need for repeated biopsies, alongside the location of the lesion in the uncinate process. Furthermore, inaccurate diagnoses with EUS biopsy procedures tend to occur most frequently in the same tumor location.

In terms of complications associated with both biopsy methods, they are characterized by low rates, underscoring the safety of these approaches. Predominantly, complications include bleeding, inflammation or infections at the biopsy site and pancreatitis [72,73]. Notably, EUS-guided biopsies demonstrate lower incidences of complications, typically ranging from 1 to 2% [74,75]. Percutaneous biopsies have higher occurrences of complications, reaching up to 5% [76]. In this systematic review and meta-analysis, a limited number of studies reported complications. Okasha et al. [43] reported one case (1.38%) of acute epigastric pain during an EUS-guided biopsy and three cases during percutaneous biopsies, along with three incidents of tumor seeding during percutaneous biopsies and one episode of severe infection in the form of a pancreatic abscess necessitating drainage (5.6%). Chai et al. [41] reported one (0.4%) serious complication during an EUS biopsy, whereas six (0.8%) such incidents were reported during percutaneous biopsies. Finally, none of the studies reported the utilization of preventive measures, such as the use of Gelfoam during the co-axial technique, to mitigate the risk of tumor seeding.

Schick et al. [77] also examined the diagnostic performance of ^18^F-FDG PET-CT for pancreatic cancer diagnosis. This imaging test, known for its ability to detect cancer through glucose metabolism characteristics, has limitations, such as an inability to precisely locate tumors [78,79,80,81,82]. Consequently, it is recommended that it be used in conjunction with CT. Comparative analysis with EUS biopsy and abdominal US revealed similar diagnostic capabilities for pancreatic cancer diagnosis [77]. However, given its reliance on glucose levels and other limitations, further research is needed to define the role of ^18^F-FDG PET-CT in stratifying and clinically managing patients with suspected pancreatic cancer.

New techniques have been proposed to achieve optimal results from biopsies of the pancreas, some of which can be performed using EUS. One such technique is through-the-needle biopsy forceps, which utilizes a microneedle to obtain two to three tissue samples through a 19-gauge FNA needle. It seems that this technique is associated with high levels of clinical and technical sensitivity, which makes it a safe choice and potential alternative in the future [83,84,85].

However, the comparison between these two biopsy techniques remains inconclusive and warrants further investigation for a comprehensive understanding. Further investigation is required of many aspects related to this comparison; for example, long-term follow-up studies are required for a better understanding of the results of each intervention. Studies also need to be conducted in specific subgroups of patients or of specific characteristics of pancreatic cancers, such as those related to the age of the patient or the location of the tumor. In addition, it is worth further studying how the experience of the doctors who perform the operation affects the results as well as evaluating patient-reported outcomes, including pain, anxiety, and satisfaction. Finally, it is also important to carry out a comparative cost-effectiveness analysis to investigate the cost-effectiveness of percutaneous and EUS-guided biopsy techniques. The choice of the appropriate method for biopsy of the pancreas is a matter of great clinical importance which concerns many medical specialties. In particular, further investigation to improve the methods and their diagnostic accuracy requires the participation of both radiologists and gastroenterologists, while at the same time it is a major issue which affects the surgical field, also requiring its participation. However, to the best of our knowledge, there is currently no published protocol outlining the further investigation of these issues.

Certain limitations of our meta-analysis should also be acknowledged. They mostly concern the heterogeneity of the included studies, since we included both RCTs and observational studies, while heterogeneity also exists in terms of the populations under study. Also, a limitation is the relatively small numbers of biopsy samples. Notably, the confounding factors inherent in ORCS were not thoroughly assessed, which may have influenced the interpretation of the results. Furthermore, the absence of subgroup analyses raises questions about the potential variability within the different populations or contexts. Additionally, the potential influences of various confounding variables, such as age and sex, across the included studies indicates the need for further investigation. The choice of different equipment in each study is another limitation, as the quality of the equipment can affect diagnostic accuracy. Another limitation is the preferential selection of the percutaneous approach for larger and consequently more readily accessible tumors, with the method therefore exhibiting higher diagnostic accuracy. Conversely, EUS is often chosen for smaller tumors, which inherently poses challenges and limits the success of the procedure.

However, a major strength of this systematic review and meta-analysis is its uniqueness as the only meta-analysis, to our knowledge, comparing these two biopsy techniques. Additionally, another strength lies in the rigorous methods employed for study selection, data extraction, and statistical analysis. This systematic review and meta-analysis represents a significant advancement in the literature regarding the identification of the optimal biopsy technique for pancreatic cancer. Our findings not only confirm the efficacy of both biopsy approaches, but also elucidate their respective strengths and weaknesses. Specifically, we observed a notable advantage favoring the percutaneous method, suggesting its potential as a preferred diagnostic tool for pancreatic cancer cases. However, the selection bias favoring larger tumors means that caution is recommended regarding the findings’ influence on clinical decision-making. Overall, the percutaneous approach should be chosen for larger tumors in clinical practice, whereas the endoscopic approach can be considered regardless of tumor size. However, these conclusions also hinge on factors such as the clinical expertise of the hospital’s healthcare staff and the availability of equipment. Through this comprehensive analysis, our aim is to provide clinicians and researchers with valuable insights to help inform decision-making processes and ultimately enhance patient outcomes in the diagnosis and management of pancreatic cancer.

## 5. Conclusions

In conclusion, pancreatic cancer stands as one of the most prevalent and perilous malignancies affecting the human body. The appropriate treatment is surgery, while the diagnosis of cancer can employ many methods. Of these, the endoscopic ultrasound approach or the percutaneous approach under US or CT guidance are preferred in daily practice. This systematic review and meta-analysis demonstrated the statistical superiority of the percutaneous approach over EUS. Nevertheless, the inherent limitations of the studies included in this meta-analysis preclude the derivation of a definitive conclusion. Without any doubt, medical expertise and skills are imperative for each method’s success. However, the selection of the biopsy method is multifaceted and is influenced by various factors, including individual patient and tumor characteristics, clinical capabilities and other pertinent considerations.

## Figures and Tables

**Table 1:** Characteristics of the included studies. Abbreviations: ORCS—observational retrospective cohort study, RCT—randomized controlled trial.

Study ID	Location	Total Tissue Samples (EUS/CT or US)	Type of Research	Type of Biopsy Method	Diagnostic Accuracy of EUS-Guided Biopsy (% [95% CI])	Diagnostic Accuracy of Percutaneous Biopsy (% [95% CI])
Mallery et al., 2002 [39]	Boston, Massachusetts, USA	149 (68/70)	ORCS	EUS-FNA and CT or US-guided FNA	76 [64.6, 85.9]	81 [70.3, 89.7]
Volmar et al., 2005 [37]	Durham, North Carolina, USA	1050 (843/207)	ORCS	EUS-FNA and CT or US-guided FNA	86.5 [83.5, 89.5]	81.7 [74.3, 89.2] for US and 82 [71.4, 92.6] for CT
Chai et al., 2023 [41]	Hangzhou, China	1074 (275/799)	ORCS	EUS-FNA and US-CNB/FNA	89.8	95.2
Horwhat, et al., 2006 [38]	Washington, DC, USA	84 (41/43)	RCT	EUS-FNA and CT or US-guided FNA	-	-
Okasha et al., 2013 [43]	Egypt	197 (72/125)	ORCS	EUS-FNA and US-FNA	88.9	87.2
Sur et al., 2014 [40]	Suwon, South Korea	106 (70/36)	ORCS	EUS-FNA and US-CNB/FNA	73	87.1
Qian et al., 2003 [42]	Boston, Massachusetts, USA	137 (51/84)	ORCS	EUS-FNA and CT- guided FNA	60.9	75.6
Erturk et al., 2005 [36]	Boston, Massachusetts, USA	70 (27/43)	ORCS	EUS-FNA and CT-guided FNA	87.5	81.7

**Table 2:** Demographic characteristics and tumor localization in pancreatic cancer patients.

	Mallery et al., 2002 [39]		Chai et al., 2023 [41]		Horwhat, et al., 2006 [38]		Sur et al., 2014 [40]	
	EUS	US/CT	EUS	US/CT	EUS	US/CT	EUS	US/CT
Age (year)	65 (35–86)	65 (28–83)	61.81 ± 0.62	63.04 ± 0.36	58	53	60.46 ± 11.96	63.97 ± 12.55
Male %	54	50	64.0	61.1	62 ± 13	67 ± 15	50	30.56
Lesion diameter	2.6 ± 0.1	3.4 ± 0.2	-	-	2.87 ± 1.01	2.63 ± 1.02	3.52 ± 1.44	3.66 + 1.31
Localization of lesion								
Head-uncinate process	45	37	121	379	28	29	40 (57.14)	26 (72.22)
Neck-body	-	-	75	271	3	1	19 (27.14)	6 (16.67)
Tail	-	-	76	133	0	1	11 (15.72)	4 (11.11)

**Table 3:** Diagnostic performance of percutaneous and endoscopic ultrasound-guided biopsies in pancreatic lesions.

Study ID	Sensitivity		Specificity		NPV		PPV	
	EUS	CT/US	EUS	CT/US	EUS	CT/US	EUS	CT/US
Mallery et al., 2002 [39]	74 [62.5, 84.5]	80 [68.7, 89.1]	100 [54.1, 100]	100 [39.8, 100]	27 [10.7, 50.2]	23 [6.8, 93.2]	100	100
Volmar et al., 2005 [37]	79.9 [75.5, 84.3]	77.9 [69.1, 86.7] and 78.6 [66.2, 91.0]	98.8 [97.2, 100]	100 [100, 100] and 100 [100, 100]	72.5 [66.8, 78.3]	48.6 [32.5, 64.8] 47.1 [23.3, 70.8]	99.2 (98.1, 100)	100 [100, 100] 100 [100, 100]
Okasha et al., 2013 [43]	84	85.5	100	90.4	100	94.7	73.3	76
Horwhat, et al., 2006 [38]	84 [64, 95]	62 [41, 80]	100 [100, 100]	100 [100, 100]	73 [44.9, 92.2]	50 [27.2, 72.8]	100.0 [100, 100]	100 [100, 100]
Chai et al., 2023 [41]	89.7	95.3	91.7	85.7	28.9	14.0	91.7	99.9
Sur et al., 2014 [40]	77.78	87.10	44.44	100	76.1	82.4	87.5	90.4
Qian et al., 2003 [42]	42	71	100	100	45	41	100	100
Erturk et al., 2005 [36]	85 [63.9, 94.8]	94.9 [83.1, 98.6]	100 [40, 100]	100 [81, 100]	57.1 [25, 84.1]	60 [23.1, 88.2]	100	100

## Data Availability

No new data are created.
